# 6-[(2-Hy­droxy-5-methyl­anilino)methyl­idene]-4-nitro­cyclo­hexa-2,4-dien-1-one

**DOI:** 10.1107/S241431462001384X

**Published:** 2020-10-23

**Authors:** Uwe Böhme, Sabine Fels

**Affiliations:** a Institut für Anorganische Chemie, Technische Universität Bergakademie Freiberg, Leipziger Str. 29, 09599 Freiberg, Germany; University of Aberdeen, Scotland

**Keywords:** crystal structure, Schiff base, tridentate ligand

## Abstract

The title compound, C_14_H_12_N_2_O_4_, is nearly planar with a dihedral angle between the aromatic rings of 1.41 (8)°. The phenolic O atom is deprotonated and the N atom of the azomethine unit carries the proton, thereby forming an intra­molecular N—H⋯O hydrogen bond. In the crystal, the mol­ecules form inversion dimers *via* pairwise O—H⋯O hydrogen bonds.

## Abstract 2

The title compound is nearly planar with a dihedral angle between the aromatic rings of 1.41 (8)°. The phenolic O atom is deprotonated and the N atom of the azomethine unit carries the proton, thereby forming an intra­molecular N—H⋯O hydrogen bond. In the crystal, the mol­ecules form inversion dimers *via* pairwise O—H⋯O hydrogen bonds.

## Graphical abstract

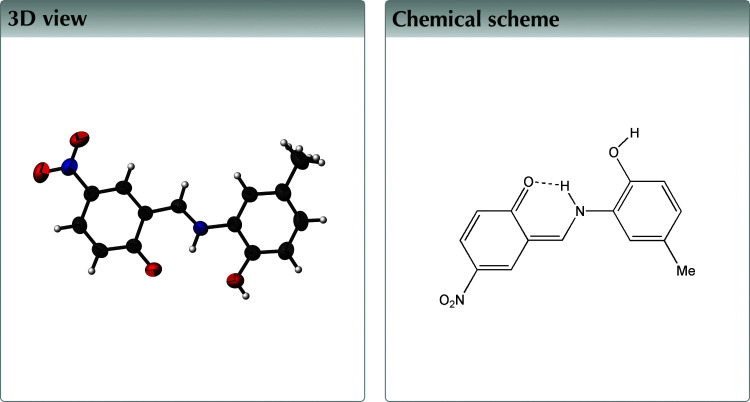

## Structure description

Aromatic Schiff bases with *ortho*-hy­droxy groups are useful as acyclic polydentate ligands for the preparation of chelate complexes with a wide variety of metal ions (Freeman & White, 1956 ▸; Calligaris & Randaccio, 1987 ▸; Pettinari *et al.*, 2001 ▸; Hernández-Molina & Mederos, 2004 ▸). We are working on silicon, tin, and titanium complexes with tridentate *O*,*N*,*O*-ligands (Böhme & Günther, 2006 ▸, 2007 ▸; Böhme *et al.*, 2006 ▸; Paul *et al.*, 2014 ▸; Warncke *et al.*, 2012 ▸, 2016 ▸; Schwarzer *et al.*, 2018 ▸).

The title compound was prepared in order to extend the series of available ligands. Its preparation was performed according to methods described in the literature for the parent compound salicycl­idene-*o*-amino­phenol (salopH_2_; Freeman & White, 1956 ▸; Pettinari *et al.*, 2001 ▸) by the reaction of 2-hy­droxy-5-nitro­benzaldehyde and 2-amino-4-methyl­phenol in ethanol.

**Figure 1:**
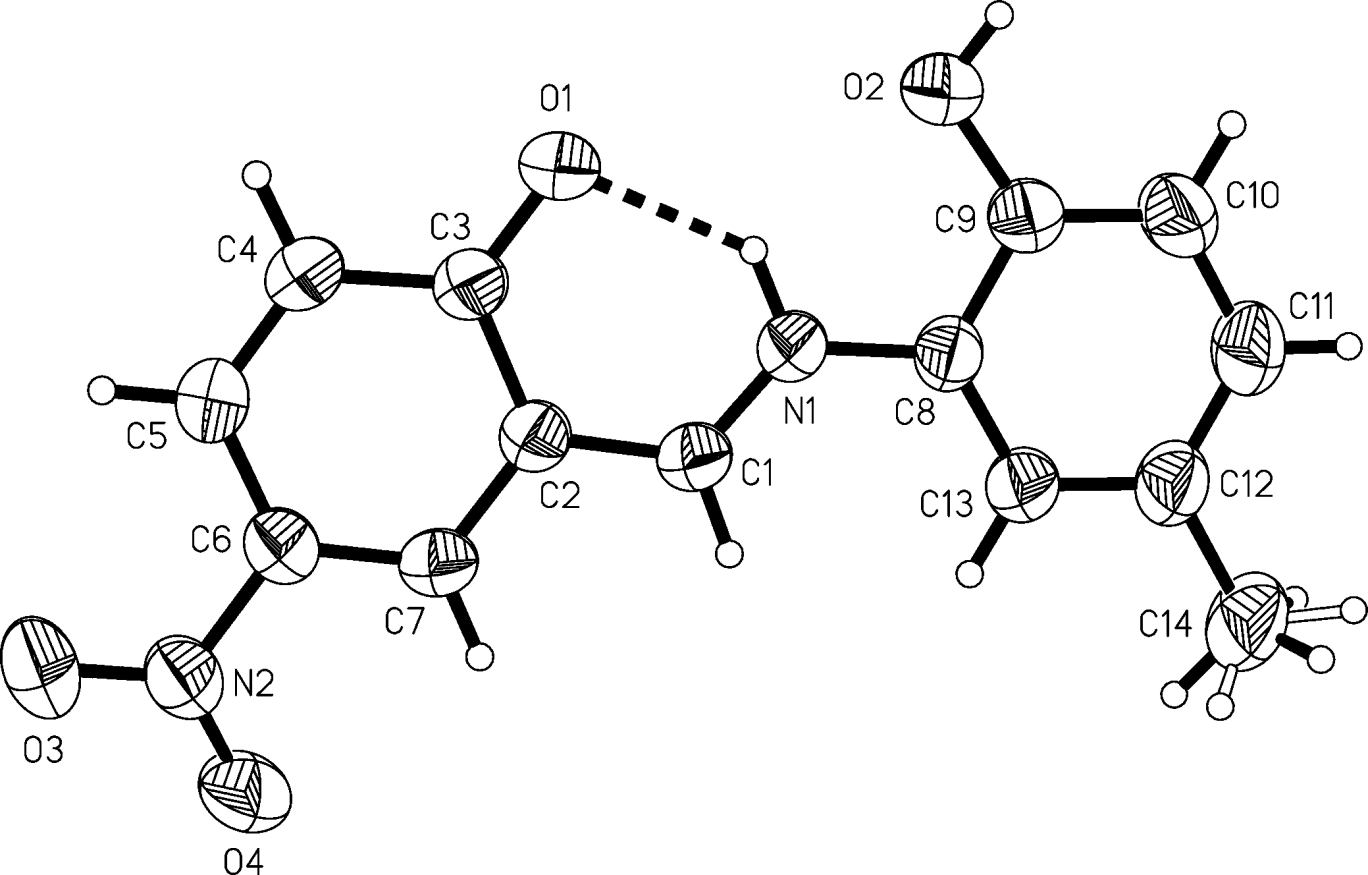
The mol­ecular structure of the title compound, drawn with 50% probability displacement ellipsoids.

The mol­ecule is nearly planar with a dihedral angle between the aromatic rings of 1.41 (8)°. Atom H2 forms an intra­molecular hydrogen bond (Table 1 ▸, Fig. 1 ▸) between the phenolic oxygen atom O1 and N1 of the azomethine unit: the hydrogen atom is localized at a distance of 0.93 (2) Å from N1, indicating the presence of the keto–amine form. The presence of a quinoidal structure is further supported by the shortening of the bond C3—O1 to 1.2734 (19) Å and the lengthening of the adjacent C—C bonds in the phenyl ring [C2—C3 = 1.446 (2), C3—C4 = 1.420 (2) Å] (Nazır *et al.*, 2000 ▸; Warncke *et al.*, 2016 ▸). There are several structure reports of Schiff bases with an oxygen atom in the *ortho*-position where the intra­molecular bridging hydrogen atom is localized at the nitro­gen atom (*e.g.* Pradeep, 2005 ▸; Dubs *et al.*, 2000 ▸; Höpfl *et al.*, 1998 ▸; Böhme & Fels, 2008*a* ▸,*b* ▸). The stabilization of salicyl­idene-imines by ‘resonance-assisted hydrogen bonding’ has been discussed previously (Hökelek *et al.*, 2004 ▸).

In the crystal, the mol­ecule forms dimers *via* pairwise O2—H9⋯O1 hydrogen bonds. An inter­molecular C—H⋯O short contact (H⋯O = 2.32 Å) to one of the O atoms of the nitro group is also present.

## Synthesis and crystallization

To 2-amino-4-methyl­phenol (1.13 g, 9.18 mmol) dissolved in ethanol (80 ml) was added 2-hy­droxy-5-nitro­benzaldehyde (1.53 g, 9.18 mmol) in ethanol (20 ml). An orange precipitate appeared after addition. The resulting suspension was heated at reflux temperature for 2 h. The precipitate was filtered off and washed with ethanol. After drying, the product was purified by recrystallization from ethanol solution. Yellow solid (2.21 g, 88.4%, m.p. 536 K). NMR (DMSO, 300 K, TMS): ^1^H: δ = 15.76, 10.17 (*s*, OH, NH, 2H), 9.31 (*s*, CH—N, 1H), 8.59–6.86 (*m*, CH_ar_ (ar = aromatic) 6H), 2.28 (*s*, Ar—CH_3_, 3H); ^13^C: 172.8 (C3), 158.9 (C1), 148.1 (C9), 136.6 (C6), 130.4, 129.8, 129.1, 128.7, 128.6, 120.6, 118.8, 116.4, 116.3 (9 signals for aromatic C), 20.1 (C14).

## Refinement

Crystal data, data collection and structure refinement details are summarized in Table 2 ▸. The methyl group at C14 is rotationally disordered over two orientations in a 0.59 (5):0.41 (5) ratio.

## Supplementary Material

Crystal structure: contains datablock(s) I. DOI: 10.1107/S241431462001384X/hb4367sup1.cif

Structure factors: contains datablock(s) I. DOI: 10.1107/S241431462001384X/hb4367Isup2.hkl

Supporting information file. DOI: 10.1107/S241431462001384X/hb4367Isup3.cml

CCDC reference: 2038926

Additional supporting information:  crystallographic information; 3D view; checkCIF report

## Figures and Tables

**Table 1:** Hydrogen-bond geometry (Å, °)

*D*—H⋯*A*	*D*—H	H⋯*A*	*D*⋯*A*	*D*—H⋯*A*
N1—H2⋯O1	0.93 (2)	1.84 (2)	2.6065 (18)	138.1 (17)
O2—H9⋯O1^i^	0.79 (3)	1.81 (3)	2.5817 (18)	163 (3)
C1—H1⋯O4^ii^	0.93	2.32	3.220 (2)	162

Symmetry codes: (i) 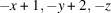 ; (ii) 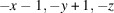 .

**Table 2:** Experimental details

Crystal data	
Chemical formula	C_14_H_12_N_2_O_4_
*M*_r_	272.26
Crystal system, space group	Monoclinic, *P*2_1_/*c*
Temperature (K)	303
*a*, *b*, *c* (Å)	6.5499 (3), 7.6232 (3), 25.6211 (11)
β (°)	96.216 (1)
*V* (Å^3^)	1271.77 (9)
*Z*	4
Radiation type	Mo *K*α
μ (mm^−1^)	0.11
Crystal size (mm)	0.47 × 0.38 × 0.12

Data collection	
Diffractometer	Bruker SMART CCD
Absorption correction	–
No. of measured, independent and observed [*I* > 2σ(*I*)] reflections	11875, 2504, 1776
*R*_int_	0.023
(sin θ/λ)_max_ (Å^−1^)	0.617

Refinement	
*R*[*F* ^2^ > 2σ(*F* ^2^)], *wR*(*F* ^2^), *S*	0.041, 0.114, 1.04
No. of reflections	2504
No. of parameters	191
H-atom treatment	H atoms treated by a mixture of independent and constrained refinement
Δρ_max_, Δρ_min_ (e Å^−3^)	0.16, −0.17

Computer programs: *SMART* and *SAINT* (Bruker, 2004 ▸), *SHELXS* (Sheldrick, 2008 ▸), *SHELXL2017/1* (Sheldrick, 2015 ▸) and *ORTEP-3 for Windows* (Farrugia, 2012 ▸).

## full crystallographic data

### Crystal data

**Table:**

C_14_H_12_N_2_O_4_	*D*_x_ = 1.422 Mg m^−^^3^
*M**_r_* = 272.26	Melting point: 536 K
Monoclinic, *P*2_1_/*c*	Mo *K*α radiation, λ = 0.71073 Å
*a* = 6.5499 (3) Å	Cell parameters from 5292 reflections
*b* = 7.6232 (3) Å	θ = 2.7–30.2°
*c* = 25.6211 (11) Å	µ = 0.11 mm^−^^1^
β = 96.216 (1)°	*T* = 303 K
*V* = 1271.77 (9) Å^3^	Prism, yellow
*Z* = 4	0.47 × 0.38 × 0.12 mm
*F*(000) = 568	

### Data collection

**Table:**

Bruker SMART CCD diffractometer	1776 reflections with *I* > 2σ(*I*)
Radiation source: sealed tube	*R*_int_ = 0.023
Graphite monochromator	θ_max_ = 26.0°, θ_min_ = 2.8°
phi and ω scans	*h* = −5→8
11875 measured reflections	*k* = −9→9
2504 independent reflections	*l* = −31→27

### Refinement

**Table:**

Refinement on *F*^2^	Primary atom site location: structure-invariant direct methods
Least-squares matrix: full	Secondary atom site location: difference Fourier map
*R*[*F*^2^ > 2σ(*F*^2^)] = 0.041	Hydrogen site location: mixed
*wR*(*F*^2^) = 0.114	H atoms treated by a mixture of independent and constrained refinement
*S* = 1.04	*w* = 1/[σ^2^(*F*_o_^2^) + (0.0502*P*)^2^ + 0.2714*P*] where *P* = (*F*_o_^2^ + 2*F*_c_^2^)/3
2504 reflections	(Δ/σ)_max_ < 0.001
191 parameters	Δρ_max_ = 0.16 e Å^−^^3^
0 restraints	Δρ_min_ = −0.17 e Å^−^^3^

### Special details

**Table:**

Geometry. All esds (except the esd in the dihedral angle between two l.s. planes) are estimated using the full covariance matrix. The cell esds are taken into account individually in the estimation of esds in distances, angles and torsion angles; correlations between esds in cell parameters are only used when they are defined by crystal symmetry. An approximate (isotropic) treatment of cell esds is used for estimating esds involving l.s. planes.
Refinement. Hydrogen atoms bonded to C were positioned geometrically and allowed to ride on their parent atoms, with C—H = 0.93 Å for C*sp*^2^, and 0.96 Å for CH_3_. *U*_iso_(H) = *xU*_eq_(C), where *x* = 1.2 for C*sp*^2^ and 1.5 for CH_3_. The hydrogen atoms at N1 and O2 (H2 and H9) were located by difference Fourier synthesis and freely refined.

### Fractional atomic coordinates and isotropic or equivalent isotropic displacement parameters (Å^2^)

**Table:**

	*x*	*y*	*z*	*U*_iso_*/*U*_eq_	Occ. (<1)
O1	0.19636 (18)	0.85732 (19)	−0.05759 (5)	0.0666 (4)	
O2	0.5013 (2)	0.9258 (2)	0.05845 (5)	0.0669 (4)	
H9	0.596 (4)	0.991 (3)	0.0647 (10)	0.093 (9)*	
N1	0.1333 (2)	0.79690 (18)	0.03950 (5)	0.0468 (4)	
H2	0.215 (3)	0.835 (3)	0.0143 (8)	0.077 (6)*	
N2	−0.5557 (2)	0.5515 (2)	−0.11991 (6)	0.0576 (4)	
O3	−0.6215 (2)	0.5556 (2)	−0.16650 (6)	0.0845 (5)	
O4	−0.6495 (2)	0.48027 (19)	−0.08672 (6)	0.0740 (4)	
C1	−0.0438 (2)	0.7351 (2)	0.02029 (6)	0.0481 (4)	
H1	−0.133979	0.696628	0.043493	0.058*	
C2	−0.1063 (2)	0.7235 (2)	−0.03427 (6)	0.0447 (4)	
C3	0.0234 (2)	0.7870 (2)	−0.07240 (6)	0.0479 (4)	
C4	−0.0523 (3)	0.7664 (3)	−0.12613 (7)	0.0563 (5)	
H4	0.027853	0.804716	−0.151711	0.068*	
C5	−0.2369 (3)	0.6928 (2)	−0.14126 (7)	0.0541 (5)	
H5	−0.282485	0.681930	−0.176767	0.065*	
C6	−0.3600 (2)	0.6328 (2)	−0.10331 (7)	0.0471 (4)	
C7	−0.2971 (2)	0.6483 (2)	−0.05097 (7)	0.0478 (4)	
H7	−0.381007	0.608690	−0.026386	0.057*	
C8	0.2130 (2)	0.8135 (2)	0.09270 (6)	0.0456 (4)	
C9	0.4090 (3)	0.8863 (2)	0.10173 (7)	0.0510 (4)	
C10	0.4949 (3)	0.9106 (3)	0.15292 (7)	0.0634 (5)	
H10	0.624875	0.960150	0.159624	0.076*	
C11	0.3879 (3)	0.8614 (3)	0.19404 (7)	0.0684 (6)	
H11	0.446815	0.879703	0.228288	0.082*	
C12	0.1941 (3)	0.7851 (3)	0.18568 (7)	0.0599 (5)	
C13	0.1080 (3)	0.7624 (2)	0.13445 (6)	0.0526 (4)	
H13	−0.021705	0.712381	0.127863	0.063*	
C14	0.0811 (4)	0.7261 (4)	0.23108 (8)	0.0869 (7)	
H14A	0.169804	0.653676	0.254283	0.130*	0.59 (5)
H14B	−0.038512	0.660356	0.217905	0.130*	0.59 (5)
H14C	0.040213	0.826966	0.249852	0.130*	0.59 (5)
H14D	0.141816	0.780054	0.262897	0.130*	0.41 (5)
H14E	0.090280	0.600890	0.234441	0.130*	0.41 (5)
H14F	−0.060592	0.760054	0.224701	0.130*	0.41 (5)

### Atomic displacement parameters (Å^2^)

**Table:**

	*U*^11^	*U*^22^	*U*^33^	*U*^12^	*U*^13^	*U*^23^
O1	0.0548 (7)	0.0944 (10)	0.0521 (8)	−0.0311 (7)	0.0125 (6)	−0.0061 (7)
O2	0.0594 (8)	0.0874 (10)	0.0547 (8)	−0.0275 (8)	0.0103 (6)	0.0008 (7)
N1	0.0490 (8)	0.0530 (9)	0.0390 (8)	−0.0094 (7)	0.0082 (6)	−0.0016 (6)
N2	0.0509 (8)	0.0630 (10)	0.0572 (10)	−0.0064 (7)	−0.0016 (7)	−0.0013 (8)
O3	0.0695 (9)	0.1175 (13)	0.0616 (10)	−0.0193 (8)	−0.0147 (7)	0.0000 (8)
O4	0.0583 (8)	0.0872 (10)	0.0757 (10)	−0.0265 (7)	0.0040 (7)	0.0075 (8)
C1	0.0475 (9)	0.0534 (10)	0.0446 (10)	−0.0096 (8)	0.0108 (7)	−0.0013 (8)
C2	0.0462 (9)	0.0476 (10)	0.0411 (9)	−0.0056 (7)	0.0074 (7)	−0.0032 (7)
C3	0.0466 (9)	0.0539 (10)	0.0446 (9)	−0.0076 (8)	0.0107 (7)	−0.0055 (8)
C4	0.0561 (10)	0.0722 (12)	0.0427 (10)	−0.0119 (9)	0.0145 (8)	−0.0019 (9)
C5	0.0567 (10)	0.0636 (12)	0.0414 (10)	−0.0032 (9)	0.0036 (8)	−0.0037 (8)
C6	0.0439 (8)	0.0481 (10)	0.0487 (10)	−0.0033 (7)	0.0021 (7)	−0.0025 (8)
C7	0.0453 (9)	0.0518 (10)	0.0476 (10)	−0.0073 (7)	0.0111 (7)	0.0024 (8)
C8	0.0514 (9)	0.0459 (9)	0.0393 (9)	−0.0042 (7)	0.0044 (7)	−0.0007 (7)
C9	0.0520 (9)	0.0543 (11)	0.0470 (10)	−0.0077 (8)	0.0064 (8)	0.0025 (8)
C10	0.0543 (10)	0.0788 (14)	0.0546 (12)	−0.0118 (10)	−0.0058 (8)	0.0012 (10)
C11	0.0759 (13)	0.0842 (15)	0.0423 (11)	−0.0087 (11)	−0.0059 (9)	0.0016 (10)
C12	0.0717 (12)	0.0652 (12)	0.0428 (10)	−0.0087 (10)	0.0062 (8)	0.0012 (9)
C13	0.0566 (10)	0.0566 (11)	0.0452 (10)	−0.0116 (8)	0.0081 (8)	−0.0013 (8)
C14	0.1101 (18)	0.1055 (18)	0.0469 (12)	−0.0257 (15)	0.0171 (12)	0.0029 (12)

### Geometric parameters (Å, º)

**Table:**

O1—C3	1.2734 (19)	C6—C7	1.365 (2)
O2—C9	1.353 (2)	C7—H7	0.9300
O2—H9	0.79 (3)	C8—C13	1.389 (2)
N1—C1	1.298 (2)	C8—C9	1.394 (2)
N1—C8	1.411 (2)	C9—C10	1.383 (3)
N1—H2	0.93 (2)	C10—C11	1.379 (3)
N2—O3	1.225 (2)	C10—H10	0.9300
N2—O4	1.2280 (18)	C11—C12	1.392 (3)
N2—C6	1.446 (2)	C11—H11	0.9300
C1—C2	1.416 (2)	C12—C13	1.382 (2)
C1—H1	0.9300	C12—C14	1.513 (3)
C2—C7	1.399 (2)	C13—H13	0.9300
C2—C3	1.446 (2)	C14—H14A	0.9600
C3—C4	1.420 (2)	C14—H14B	0.9600
C4—C5	1.351 (2)	C14—H14C	0.9600
C4—H4	0.9300	C14—H14D	0.9600
C5—C6	1.405 (2)	C14—H14E	0.9600
C5—H5	0.9300	C14—H14F	0.9600
			
C9—O2—H9	112.4 (18)	C13—C8—N1	123.79 (15)
C1—N1—C8	128.37 (14)	C9—C8—N1	115.70 (14)
C1—N1—H2	114.1 (13)	O2—C9—C10	125.12 (16)
C8—N1—H2	117.6 (13)	O2—C9—C8	115.95 (15)
O3—N2—O4	122.47 (16)	C10—C9—C8	118.92 (15)
O3—N2—C6	118.86 (15)	C11—C10—C9	119.98 (17)
O4—N2—C6	118.67 (15)	C11—C10—H10	120.0
N1—C1—C2	123.24 (14)	C9—C10—H10	120.0
N1—C1—H1	118.4	C10—C11—C12	121.78 (18)
C2—C1—H1	118.4	C10—C11—H11	119.1
C7—C2—C1	118.74 (14)	C12—C11—H11	119.1
C7—C2—C3	120.10 (14)	C13—C12—C11	118.04 (17)
C1—C2—C3	121.17 (14)	C13—C12—C14	120.65 (18)
O1—C3—C4	122.77 (14)	C11—C12—C14	121.31 (18)
O1—C3—C2	120.57 (15)	C12—C13—C8	120.75 (16)
C4—C3—C2	116.67 (14)	C12—C13—H13	119.6
C5—C4—C3	122.11 (15)	C8—C13—H13	119.6
C5—C4—H4	118.9	C12—C14—H14A	109.5
C3—C4—H4	118.9	C12—C14—H14B	109.5
C4—C5—C6	119.96 (16)	H14A—C14—H14B	109.5
C4—C5—H5	120.0	C12—C14—H14C	109.5
C6—C5—H5	120.0	H14A—C14—H14C	109.5
C7—C6—C5	121.13 (15)	H14B—C14—H14C	109.5
C7—C6—N2	119.33 (15)	C12—C14—H14D	109.5
C5—C6—N2	119.53 (15)	C12—C14—H14E	109.5
C6—C7—C2	120.03 (14)	H14D—C14—H14E	109.5
C6—C7—H7	120.0	C12—C14—H14F	109.5
C2—C7—H7	120.0	H14D—C14—H14F	109.5
C13—C8—C9	120.51 (15)	H14E—C14—H14F	109.5
			
C8—N1—C1—C2	−179.40 (16)	C1—C2—C7—C6	−178.99 (16)
N1—C1—C2—C7	177.42 (16)	C3—C2—C7—C6	0.7 (3)
N1—C1—C2—C3	−2.3 (3)	C1—N1—C8—C13	0.4 (3)
C7—C2—C3—O1	179.29 (16)	C1—N1—C8—C9	−179.56 (17)
C1—C2—C3—O1	−1.0 (3)	C13—C8—C9—O2	177.63 (16)
C7—C2—C3—C4	−0.8 (2)	N1—C8—C9—O2	−2.4 (2)
C1—C2—C3—C4	178.95 (16)	C13—C8—C9—C10	−1.5 (3)
O1—C3—C4—C5	−179.43 (18)	N1—C8—C9—C10	178.44 (16)
C2—C3—C4—C5	0.6 (3)	O2—C9—C10—C11	−178.40 (19)
C3—C4—C5—C6	−0.4 (3)	C8—C9—C10—C11	0.7 (3)
C4—C5—C6—C7	0.4 (3)	C9—C10—C11—C12	0.7 (3)
C4—C5—C6—N2	−178.90 (16)	C10—C11—C12—C13	−1.2 (3)
O3—N2—C6—C7	171.96 (17)	C10—C11—C12—C14	177.9 (2)
O4—N2—C6—C7	−8.3 (2)	C11—C12—C13—C8	0.4 (3)
O3—N2—C6—C5	−8.8 (3)	C14—C12—C13—C8	−178.81 (19)
O4—N2—C6—C5	170.98 (16)	C9—C8—C13—C12	1.0 (3)
C5—C6—C7—C2	−0.5 (3)	N1—C8—C13—C12	−178.96 (17)
N2—C6—C7—C2	178.75 (15)		

### Hydrogen-bond geometry (Å, º)

**Table:**

*D*—H···*A*	*D*—H	H···*A*	*D*···*A*	*D*—H···*A*
N1—H2···O1	0.93 (2)	1.84 (2)	2.6065 (18)	138.1 (17)
O2—H9···O1^i^	0.79 (3)	1.81 (3)	2.5817 (18)	163 (3)
C1—H1···O4^ii^	0.93	2.32	3.220 (2)	162

Symmetry codes: (i) −*x*+1, −*y*+2, −*z*; (ii) −*x*−1, −*y*+1, −*z*.
